# Differentiation of Human Breast-Milk Stem Cells to Neural Stem Cells and Neurons

**DOI:** 10.1155/2014/807896

**Published:** 2014-11-25

**Authors:** Seyed Mojtaba Hosseini, Tahere Talaei-khozani, Mahsa Sani, Bahareh Owrangi

**Affiliations:** ^1^Student Research Committee, Shiraz University of Medical Sciences, Shiraz, Iran; ^2^Cell and Molecular Student Research Group, Medical Faculty, Shiraz University of Medical Sciences, Shiraz, Iran; ^3^Stem Cell Laboratory, Department of Anatomy, Shiraz University of Medical Sciences, Shiraz, Iran; ^4^Department of Anatomy, Shiraz University of Medical Sciences, Shiraz, Iran

## Abstract

*Objectives.* Human breast milk contains a heterogeneous population of cells that have the potential to provide a noninvasive source of cells for cell therapy in many neurodegenerative diseases without any ethical concern. The objectives of this study were to differentiate the breast milk-derived stem cells (BMDSC) toward neural stem cells and then into the neurons and neuroglia. *Materials and Methods.* To do this, the BMDSC were isolated from human breast milk and cultured in Dulbecco's modified Eagle medium/F12 (DMEM/F12) containing fibroblast growth factor (bFGF). The cells were then characterized by evaluation of the embryonic and stem cell markers. Then, the cells were exposed to culture medium containing 1% B27 and 2% N2 for 7–10 days followed by medium supplemented with B27, N2, bFGF 10 *µ*g/mL, and endothelial growth factor (EGF) 20 *µ*g/mL. Then, the sphere-forming assay was performed. The spheres were then differentiated into three neural lineages by withdrawing growth factor in the presence of 5% FBS (fetal bovine serum). The immunofluorescence was done for *β*-tubulin III, O4, and GFAP (glial fibrillary acidic protein). *Results.* The results indicated that the cells expressed both embryonic and mesenchymal stem cell (MSC) markers. They also showed neurospheres formation that was nestin-positive. The cells were also differentiated into all three neural lineages. *Conclusion.* The BMDSC can behave in the same way with neural stem cells. They were differentiated into oligodendrocytes, and astrocytes as well as neurons.

## 1. Introduction

Increase in the prevalence of neurodegenerative diseases and neural damage has caused the scientists to search for various approaches such as cell therapy to improve the neurogenesis techniques [1]. A lot of investigations confirmed the presence of adult stem cells in various tissues including breast milk [2]. Breast milk contains a heterogeneous cell population; besides, a subpopulation with stem cell properties including the ability to be differentiated into different cell lineages has been isolated from fresh human milk [2, 3]. A bipotential stem cell with the differentiation capacity into mammary epithelial cell and myoepithelial cells has been detected in mammary gland tissue [4]. Maternal mammary stem cells have been considered as one of the cell sources in breast milk [5]. The presence of exfoliated epithelial cells from alveoli, macrophages, and lymphocytes was also reported [6, 7]. It has been also reported that 10–15% of the cells isolated from fresh breast milk expressed mesenchymal stem cell (MSC) markers and culturing the isolated cells led to an increase in the MSC population due to their higher capacity of cell proliferation [2]. The presence of a nestin-positive subpopulation was also reported in the breast milk-derived cells; however, the frequency of these cells was low [8, 9].

Previous studies identified the presence of activated mammary stem cell (MaSC) in breast milk through staining cells for MaSC markers [2]. Hassiotou et al. named these cells as human breast-milk stem cells (hBSCs) [3]. Their results demonstrated that these stem cells have a capability to be differentiated into mammary cells (luminal and myoepithelial). Furthermore, they determined the expression of a group of embryonic stem cell- (ESC-) associated genes such as OCT4, KLF4, NANOG, and SOX2 in hBSCs [3]. Also, it has been reported that the phenotype, colony morphology, and differentiating capability of hBSCs are similar to those in ESC [3, 10]. Consequently, as breast milk contains these special pluripotent stem cells, it can be utilized as a valuable and a new easily available source for regenerative medicine [3, 10]. The presence of nestin-positive cell population and also a subpopulation of expressed ESC markers in breast milk caused this noninvasive source of stem cells to be considered as a good candidate for differentiation into neural cell lineage [10].

Both mammary gland and nervous system have the same origin. It has been demonstrated that the common regulators play role in the development of both mammary gland and neuroepithelium, and these regulators are also involved in ESCs differentiation and self-renewal [11]. These common embryonic origins and also common regulators may suggest breast milk-derived cells as a good source for neural cell lineages differentiation.

Previous studies reported that neural stem cells showed the capability to be differentiated toward neuronal cells [12, 13]. They may be considered as an appropriate source for cell replacement therapies (CRTs) of the brain diseases [14]. However, there have been various reports which show the risks of neural stem cell application for the patients including tumor formation, insufficient migration, immune rejection, surgical threats, and transmission of infections possibility during transplantation [15]. Besides, neural stem cell isolation is invasive. Therefore, finding an alternative stem cell source is essential to overcome such issues and barriers.

The pluripotency of the breast milk-derived cells was evaluated by exposing the cells to various culture conditions. The cells treated with neurogenic medium expressed nestin and tubulin which indicated the cell differentiation into neural progenitor cell and neuron-like cells, respectively [3]. This study tried to find the breast milk-derived cell ability to be differentiated into three neural cell lineages, neurons, astrocytes, and oligodendrocytes. Also, the current study showed that the breast milk-derived cell behaved in a similar way to neural stem cells in vitro.

## 2. Materials and Methods

### 2.1. Sample Collection

This study was approved by the Ethics Committee of Shiraz University of Medical Sciences, and all participants provided informed written consent. Women, who were healthy breastfeeding participants, submitted informed written consent prior to sample collection. In aseptic procedures, mature breast milk (5–200 mL) samples were collected from them and instantly transferred to the stem cell laboratory.

### 2.2. Isolation of Cells from Breast Milk Sample

Disinfected phosphate buffered saline (PBS) (pH 7.4, Gibco) equal volumes were used to dilute the breast milk and they were centrifuged for 20 minutes at 20°C at 805 g. Then, skim milk liquid component and fat layer were taken out. In PBS, the cell pellet, the remaining part, was washed three times. After that, the cell pellet was resuspended in 10% fetal bovine serum (FBS, Certified, Invitrogen) within PBS (blocking buffer). To determine each sample's cell viability and cell concentration, the Neubauer hemocytometer was utilized with Trypan Blue for segregation.

### 2.3. Culture of the Isolated Cells

Breast milk cells (40000 cells/mL) were cultured in plates coated with gelatin. They were incubated at 5% CO_2_ and 37°C; also, the media were changed daily. After 5 days of culturing, each colony was separately selected and relocated in new dishes and cultured in medium in order to make feeder culture of the second and third ones. Furthermore, ultralow binding plates were used to seed cells on them to have spheroid culture and the ES media (DMEM/F12, KOSR (knockout serum replacement) 10%, nonessential amino acid 1%, bFGF 10 *μ*g/mL, and pen/strep 1%) were added to the plates. Whenever the cells needed to be passaged, they were trypsinized (Gibco) at 37°C in 5 minutes and were divided to 1 : 2.

### 2.4. Immunocytochemistry for the Breast Milk-Derived Stem Cells

Some embryonic and mesenchymal stem cell markers antibodies such as Nanog, OCT4 (Abcam ab19857 1 : 500), SOX2, CD44 (Abcam ab119863 1 : 500), CD105 (Abcam ab44967 1 : 250), CD106 (Abcam ab19569 1 : 500), CD90 (Abcam ab225 1 : 250), and CD133 (Millipore MAB4399) were used to characterize the isolated cells from human breast milk by immunocytochemistry method. Briefly, the cultured cells were fixed by paraformaldehyde 4% in 4°C for 20 minutes; after washing with PBS, primary antibodies were diluted in PBS containing 0.3% triton and 5% goat serum and the samples were kept in room temperature for 60 minutes. After that, washing with PBS was performed for 3 times. Then secondary antibodies were added and, after 45 minutes of incubation in room temperature, the last washing with PBS was done.

### 2.5. Differentiation into Neural Stem Cell

The isolated cells were cultured in gelatin coated plates with concentration of 10000 cells/cm^2^ with DMEM/F12, 1% B27, and 2% N2 for 7–10 days. After this period of time, some sphere-like cell aggregations were seen. These spheres were separated and passaged in a different plate using DMEM/F12 containing B27, N2, bFGF 10 *μ*g/mL, and EGF 20 *μ*g/mL (NS-A media).

### 2.6. Sphere-Forming Assay

The differentiated cells were cultured in appropriate media (NS-A media). The media were removed every 3–5 days and changed with the fresh ones. The cells were incubated in 5% CO_2_ at 37°C for 7 days. Then, the spheres were formed and each cell of these spheres was capable of providing another sphere by passaging. For neural stem cells identification, immunocytochemistry was performed for nestin antibody (Millipore, AB5922) on neurospheres and CD133 (Millipore MAB4399) [16] for neural stem cells with protocol mentioned above.

### 2.7. Neural Stem Cell Differentiation

Neural stem cells (NSCs) (5000 cells/mL) were seeded in poly-L-ornithine-coated culture dishes to be differentiated into neuron, oligodendrocyte, and astrocyte. For differentiation, bFGF and EGF were removed from the media and FCS 5% was added to them and after 5 days the differentiated neural stem cells provided neurons, oligodendrocytes, and astrocytes. For neuron, oligodendrocyte, and astrocyte detection, antibodies, *β*-tubulin III (Promega G7121, 1 : 2000), O4 [17] (Millipore, MAB345 1 : 50), and GFAP [18] (DakoCytomation, Code number Z0334 1 : 500), respectively, were assessed by an immunocytochemistry method similar to the one previously described.

### 2.8. 4′,6-Diamino-2-phenylindole Dihydrochloride (DAPI) Staining

To detect the cells' nucleus, they were fixed with paraformaldehyde 4% and then 4′,6-diamino-2-phenylindole dihydrochloride (Millipore S7113 1 : 1000) was added to the fixed cells and they were kept in room temperature for 30 minutes.

## 3. Results

### 3.1. Breast-Milk Stem Cell Isolation and Characterization

**Figure 1:**
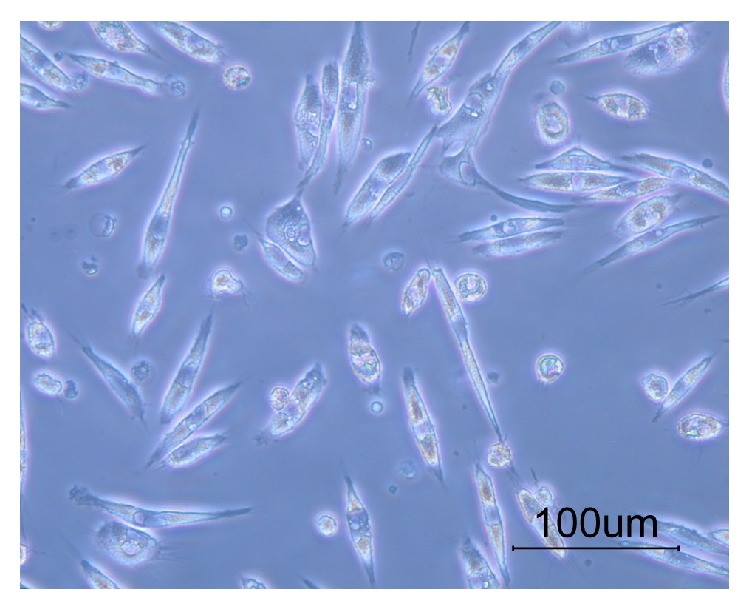
The breast milk-derived cells showed a fusiform morphology with few processes.

The isolated breast-milk-derived cells were adherent to the plates and their morphology was similar to the cells of myoepithelium of the breast (Figure 1). They were cultured to the 10th passage and after each passage the provided cells were capable of proliferating and kept their expansion potential.

**Figure 2:**
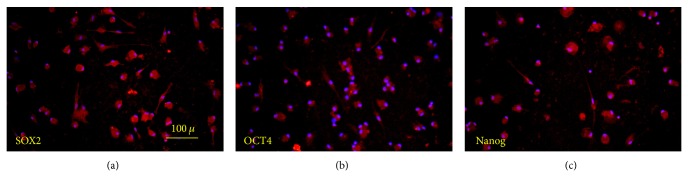
The immunofluorescence of the cells showed that a subpopulation of the breast milk-derived cells reacted for OCT4, Nanog, and SOX2 antibodies.

The immunohistochemistry staining showed a subpopulation of breast milk-derived cells that expressed embryonic stem cells markers such as Nanog (66.2% ± 6.52), OCT4 (42.9% ± 6.99), and Sox2 (57.3% ± 7.74) (Figure 2). A subpopulation of the isolated cells also expressed markers that were also detected on the surface of mesenchymal stem cells including CD44 (67% ± 10.7), CD105 (68.3% ± 3.91), CD106 (8.4% ± 2.36), and CD133 (2.76% ± 1.93).  Table 1 summarizes the frequency of the positive cell for each marker.

### 3.2. The Neural Stem Cells Differentiated from Breast Milk-Derived Cells

**Figure 3:**
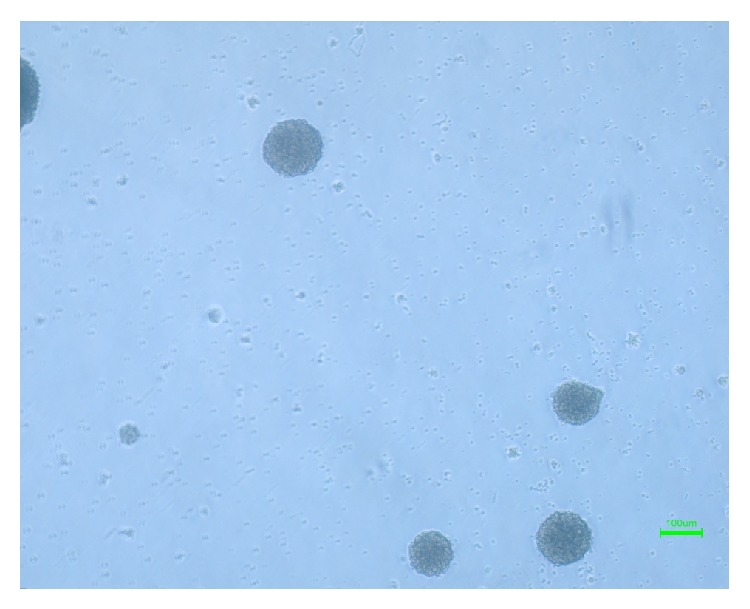
Neurospheres, formed after exposing the breast milk-derived cells to neurogenic medium.

After exposing the breast-milk stem cells to NS-A media (DMEM/F12, N2, B27, bFGF, and EGF) for 5 days, they formed shiny, floating, sphere-like cell aggregations, neurospheres, measuring about 100 *μ*m in diameter (Figure 3). The enzymatically dissociated neurospheres showed the capability of forming a new sphere within 5–7 days. In addition, the cells kept their sphere-forming ability till the 8th passage without any significant change in their sphere-forming frequency (Figure 8).

A small subpopulation of the breast milk-derived cells expressed nestin (7.4% ± 3.30) and CD133 (2.76% ± 1.93) as markers for neural stem cells. The frequency of the nestin-positive (58.20% ± 6.71) (Figures 6 and 9) and CD133-positive (58.74% ± 3.36) (Figures 7 and 10) cells was increased significantly after exposing the cells to neurogenic media and neurosphere formation. The behavior of the nestin-positive breast milk-derived cells treated with neurogenic media, including the formation of spheroid aggregates, was similar to the neural stem cells. Therefore, they may be neural stem cell-like cells.

### 3.3. Capability of Producing Three Neural Lineages

The most important characteristic for specification of neural stem cells is their ability to produce three neural lineages consisting of neurons, oligodendrocytes, and astrocytes. Differentiated neurons showed two processes extended from cell body. The cells with branched intricate processes extended from cell body represented oligodendrocytes. Differentiated astrocytes showed a flat or polygonal cell body with several processes.

**Figure 4:**
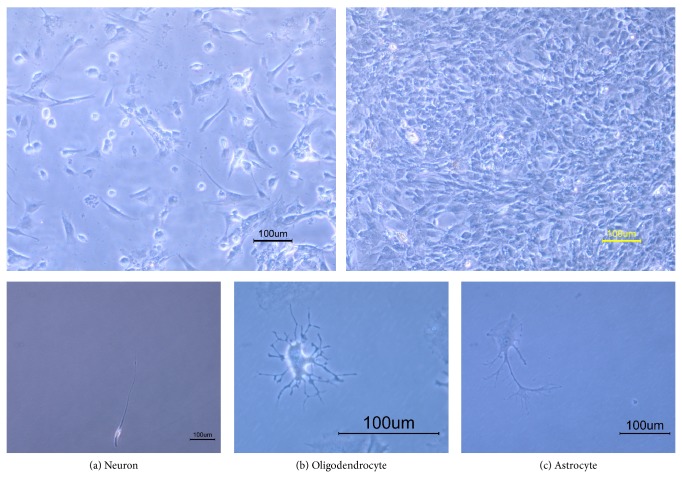
Inverted microscopy of the breast milk-derived cells differentiated toward three neural cell lineages.

After adding 5% fetal bovine serum, removing growth factors (bFGF and EGF), to the cells for 5 days, the neural stem cell-like cells proliferated and attached to the plate totally and their appearances were completely changed into three neural cell lineages (Figure 4).

**Figure 5:**
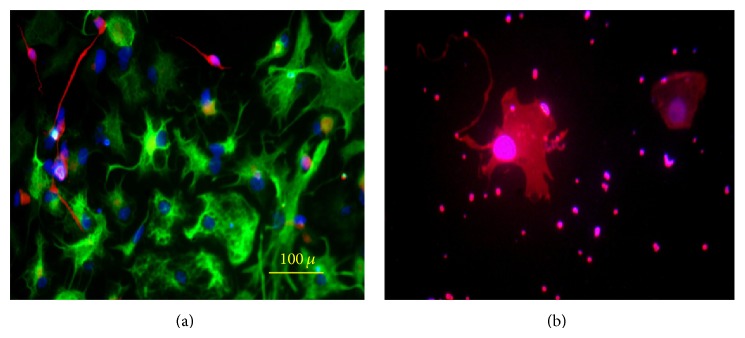
(a) Neurons and astrocytes differentiated from breast milk stem cells-derived neural stem cell stained with anti-*β*-tubulin-Alexa Flour 568 (red) and anti-GFAP-Alexa Flour 488 (green); (b) oligodendrocyte differentiated from breast milk stem cell-derived neural stem cell stained with anti-O4-Alexa Flour 568.

**Figure 6:**
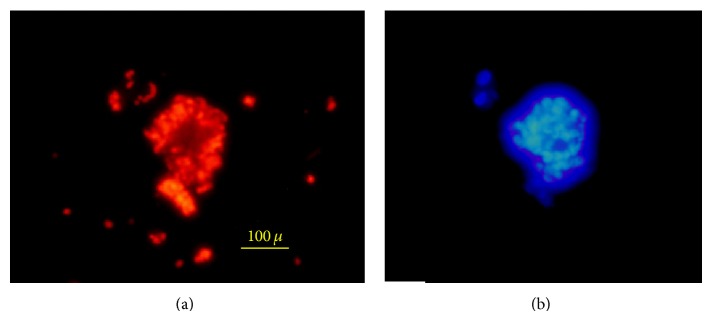
A neurosphere stained with anti-nestin-Alexa Flour 568 (red) antibody (a) and DAPI (b).

**Figure 7:**
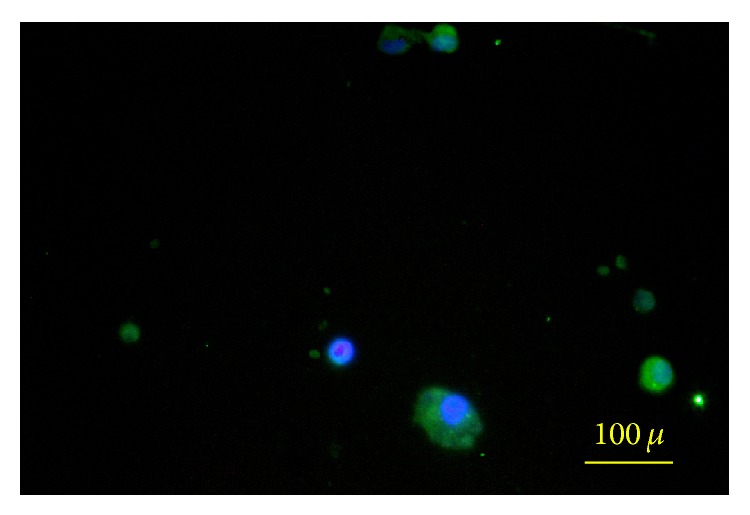
Neural stem cell stained with CD133 and DAPI.

**Figure 8:**
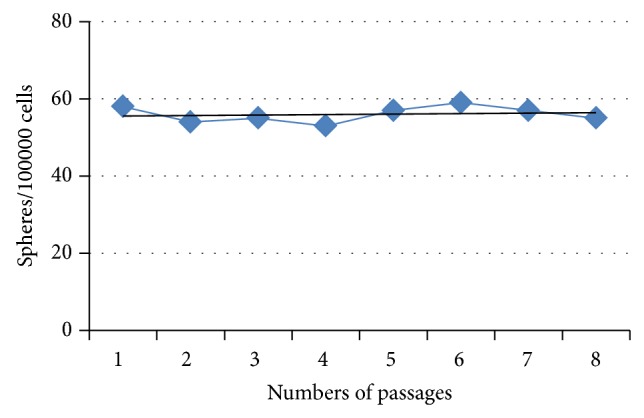
Sphere frequency assay.

**Figure 9:**
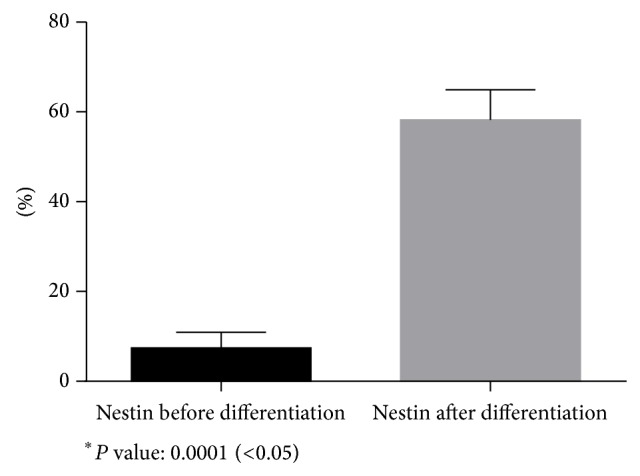
Comparison of nestin expression before differentiation and after differentiation.

**Figure 10:**
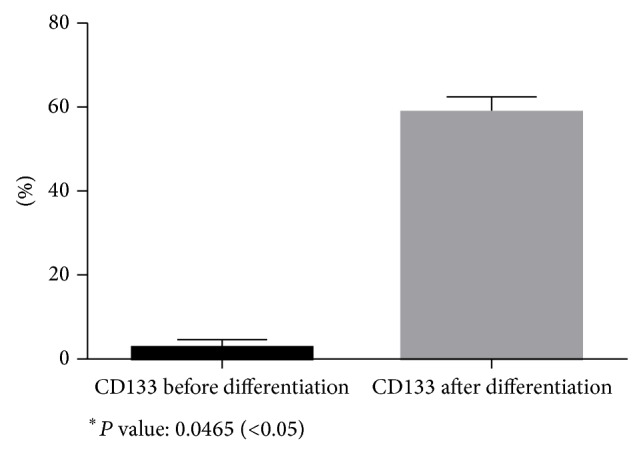
Comparison of CD133 expression before differentiation and after differentiation.

To detect neurons, the expression of neuronal marker, *β*-tubulin III, was evaluated. The percentage of the tubulin III was 19% ± 2.44 of the cells treated with neurogenic media. For detection of oligodendrocyte, anti-O4 antibody [17] was used and the frequency of the O4-positive cells was 14.31% ± 2.31; and for detection of astrocytes, anti-GFAP antibody [18] was administrated and it was shown that 66.69% ± 7.50 of the cells were astrocytes (Figure 5, Table 2).

## 4. Discussion

The presence of the cells with various origins was detected in human breast milk. Therefore, breast milk cells have been suggested to be used for treating neonatal disorders [19]. Breast-milk stem cells have been previously demonstrated to express nestin, a neuroectoderm marker; however, a few samples contained the cells expressing neurofilament, a late neural differentiation marker. However, the frequency of the nestin-positive cells was low [8]. The presence of nestin-positive cells indicated that the breast-milk stem cells can be an appropriate candidate for differentiation toward neurons or neuroglia. The results of the current study revealed that the exposure of the whole cell population of breast milk to neurogenic medium led to an increase in the presence of nestin-positive cells and that the cell behavior in neurosphere formation is the same as what happened in the culture of neural stem cells [20].

Further differentiation of nestin-positive cells into the neuron as well as neuroglia was shown in this study as indicated by the presence of cells that expressed *β*-tubulin as neuron marker, O4 as oligodendrocyte marker, and GFAP as astrocyte marker. Several studies also showed that nestin-positive cells were derived from the neurosphere of the neural stem cells isolated from the avian species [21], mouse embryo [22], adult mouse [23], and human [24] and differentiated into three neural lineages. It seems that the cells derived from breast milk were differentiated in the same way as in the case of neural stem cells.

Mesenchymal stem cells derived from human adipose tissue [25, 26], bone marrow [26], muscle [27], and umbilical cord [28] have been shown to be able to be differentiated toward neuron-like cells. Neuron-like cells differentiated from Wharton jelly of the umbilical cord were also transplanted to the A*β*PP/PS1 mouse. The transplantation improved the cognitive reactions of the mouse and decreased the level of amyloid *β*-peptides deposition [29]. Human umbilical mesenchymal stem cells-derived neurospheres with BDNF were transplanted to the injured spinal cord and the morphologic and functional recoveries have been demonstrated [30]. These data indicated that nonneural stem cells may be functional when they were transplanted to the animal models. Therefore, the neural-like cells or neuroglia derived from breast milk also may be as effective as those derived from the other sources; however, it needs further investigations.

Our results indicated that breast milk-derived stem cells express embryonic cell markers such as Nanog, OCT4, Sox2, SEEA4, and TRA 1–60/81. The expression of some mesenchymal stem cell markers has been shown previously [19]. It indicated that these cells may behave like both embryonic and mesenchymal stem cells. Embryonic stem cells have a great potential to be differentiated toward neural cell lineages [31, 32]. They can be differentiated into neural progenitor cells spontaneously [33, 34]. The stability in electrical phenotype of stem cell-derived neuron has been shown previously [35], revealing that the differentiated cells may be functional. The transplanted Mesenchymal stem cells in CNS has been shown they are capable to survive and differentiate to neural lineages [36]. The breast milk-derived stem cells showed the capability to be differentiated into neural cell lineages and their similarity to both embryonic and mesenchymal stem cells makes them a good candidate for cell therapy in neurodegenerative diseases.

## 5. Conclusion

As the isolation of neural stem cells is an invasive procedure, using breast milk-derived cells as a source of cell therapy may be preferable especially for the patients in child-bearing ages. However, the physiological test and also in vivo studies should be done to ensure that the cells do not dedifferentiate and are functional when they are transplanted.

## Figures and Tables

**Table 1:** The percentage and SD of the cells that expressed various stem cell markers.

CD44	CD105	CD90	CD106	SOX2	Nanog	OCT4/3	Nestin	CD133
67% ± 10.7	68.3% ± 3.91	19.7% ± 3.74	8.4% ± 2.36	57.3% ± 7.74	66.2% ± 6.52	42.9% ± 6.99	7.4% ± 3.30	2.76% ± 1.93

**Table 2:** The percentage of *β*-tubulin, O4, and GFAP.

*β*-Tubulin III	O4	GFAP

19% ± 2.44	14.31% ± 2.31	66.69% ± 7.50

## Abbreviations

GFAP: Glial fibrillary acidic protein
MSC: Mesenchymal stem cell
bFGF: Basic fibroblast growth factor
BMDSC: Breast milk-derived stem cells
EGF: Epidermal growth factor
Masc: Mammary gland stem cell
CNS: Central nervous system
ESC: Embryonic stem cell
CRT: Cell replacement therapy
hBSCs: Human breast-milk stem cells
iPSC: Induced pluripotent stem cell
NSC: Neural stem cell
FBS: Fetal bovine serum.
